# Case of Complicated Visceral Disease, &c. With the Appearances on Dissection

**Published:** 1819-01-01

**Authors:** Jos. Stilon

**Affiliations:** Surgeon, of His Majesty's Dock Yard, Malta


### Medico-Chirurgical Journal;

OR,

### Quarterly Register

OF

### MEDICAL AND SURGICAL SCIENCE.

Quarterly Series.] JANUARY 1, 1819. [Vol. I.

### First Department.

### PRACTICAL ESSAYS AND COMMUNICATIONS,

*Original or Translated.*

I.

### *Case of complicated Visceral Disease, &c. with the Appearances on Dissection.*

By Mr. Jos. Stilon, Surgeon, of His Majesty's Dock Yard, Malta.

ANDREW BURGIA, ætat. 33 years, of a spare and delicate constitution (a servant to Mr. C. H. Smith, Naval Officer in His Majesty's Dock Yard at Malta) complained, on the 14th of May, 1817, of severe pain in the head, back, and limbs; eyes suffused; pulse 100, hard, and full; tongue foul; bowels costive, with great thirst.

I took twenty-six ounces of blood from the arm, gave him a strong cathartic of sulph. mag. and ordered barley water as common drink during the day.

On the 15th, something better; two grains of the sub-mur. hydrarg. with half a grain of the pulv. antimon. were given three times a day.

16th. Much better; complains of pain in the scrotum, which, on examination, was found much enlarged, and painful to the touch; but, as I had frequently observed this symptom in the fever of the country, I only ordered the parts to be fomented with a decoction of camomile flowers, which soon reduced the swelling, and allayed the pain. The man continued to improve under this treatment, and on the 20th was discharged to his duty.

After a month had elapsed, the same symptoms recurred, accompanied with severe pain in the course of the colon, and a vomiting of very acrid bile. Venesection was again had recourse to; a strong cathartic of the sulph. mag. was given, which produced several fœtid and bilious stools. After the operation of the cathartic, the effervescent draughts were given every two hours, with ten drops of the tinct. opii in each. To these remedies, the vomiting soon yielded; calomel and antimony were then given, as before, and again he soon got well.

From this period, he was generally attacked with the same complaint every month, or every fortnight; the testicle became every day larger, but was free from pain, except when fever supervened. Attributing all his complaints now to the state of the testis, I inquired whether he had ever been affected with syphilis, when he assured me that he had, when about the age of eighteen, contracted a gonorrhœa, which went off, without the use of medicines, in four or five days. I, however, adopted the mercurial treatment, giving him the blue pill in doses of five grains, thrice a day. His mouth was affected three times; but the testicle increased in size, and much constitutional irritation ensued. The Surgeon of the Naval Hospital was now consulted, and he advised me to continue the treatment, and to rub on the part the ung. hydrarg. fort. This was persisted in till such a state of irritability was induced, that I was obliged to discontinue the exhibition of mercury, fully convinced of its inefficacy. Purgatives were substituted, and blisters were applied over the tumour; but the result was merely palliative. The testicle continued to increase. It was of a pyrimidal form, its base being downwards, and the apex terminating at the abdominal ring; the scrotum elastic to the touch, its surface smooth, while, at the lower and back part, a hard body could be felt, similar in size and situation to the testicle in hydrocele. That it was not the right testicle, I was convinced, as this had not descended into the scrotum, and could be distinctly felt above Poupart's ligament.

I now had it examined by several medical gentlemen, who were of opinion that it should be treated as hydrocele, as this would not interfere with a future operation, should it prove to be of a different nature. On the 9th of March, 1818, therefore, I punctured the scrotum, and drew off about four ounces of a bloody fluid, in which floated a little coagulated lymph. After the canula was withdrawn, cold lotions were applied, and the wound healed without the least inflammation. The disease still gaining ground, I, on the 15th of the same month, removed the testicle, which I found to be highly diseased, about six inches long, and large in proportion. What had been taken for the testicle, was no more than a protuberance; the substance of which had very much the appearance of tallow. The body of the testicle, though so much enlarged, had not departed greatly from its natural form. The spermatic cord was sound. On the third day, the wound was dressed; it only united partially by the first intention; and being rather indolent, a glass of good wine was daily given to the patient.

On the twenty-sixth day after the operation, the wound was perfectly healed, and nothing seemed to remain of consequence, except debility. While confined to his bed, the only medicines had been an occasional aperient.

About a month after having discontinued my visits, I was sent for, and found him in bed, complaining of pain in the abdomen; sickness at stomach; and he had, before my arrival, vomited a quantity of very acrid bile, rather of a dark colour, and passed several bilious and fœtid stools. The pulse was irregular, and he had excruciating pain in the back. I examined the abdomen, which I found much distended, and a hard tumour extending from the left hypochondrium to the right, for the space of nearly thirteen inches; and, from above downwards, between the epigastric and umbilical regions, about ten inches; it was not painful on pressure. A cathartic was ordered; and, after its operation, he felt much better, and continued so for a fortnight, when he was again taken ill in the same manner; and what appeared very remarkable, he felt much worse during the prevalence of the sirocco, and the tumour became much more enlarged than when the wind was northerly. He now began to spit a bloody membraneous substance, containing a large portion of air, producing a crepitus when pressed between the fingers. Some days he would spit up a great quantity, and then remained two or three days without spitting any. When this happened, the urine would become like curdled milk, depositing a yellowish sediment, with pieces of coagulated blood; but no sooner did the bloody expectoration return, than the urine would recover its natural clearness: and so these appearances alternated through the remaining stages of the disease. A large opiate was ordered every evening, with which he would obtain scarcely an hour or two of sleep in the four and twenty. No medicine would remain on his stomach, and he now loathed his wine, and I was obliged to dissolve his opium in water, as all sorts of spirits, even in the most minute portion, would produce violent irritation.

A medical gentleman, a native of this country, who was consulted, prescribed ass's milk, but, on trying it once, a diarrhœa ensued that almost carried him off. He died on the 26th of June, exceedingly emaciated.

### APPEARANCES AFTER DEATH.

On opening the abdomen, the first thing that presented itself to view was the liver, which was very thin and much elongated, extending down to the pubes, and covering the greatest part of the intestines. But its convex part, that was in contact with the diaphragm, was much enlarged, thickened, and, on cutting into its substance, it was found very much diseased, and had the same appearance as the testicle that was removed. My attention was now attracted to the large tumour in the left side; and being apprehensive that the spermatic cord might be diseased, I dissected it from the abdominal ring to its origin, but found it sound, and the only alteration was that occasioned by the large tumour under it, which put it on the stretch. On removing the intestines, mesentery, and omentum, six fungous bodies were seen springing from the large tumour. These had made their way through the peritonæum, and were of a reddish brown colour.

The tumour, which was taken by several medical gentlemen for an enlarged spleen, was now found to be the left kidney in a mass of disease, measuring twelve inches, as it lay across the abdomen from left to right, and ten inches from above downwards. The superior half of the kidney had not lost its natural appearance externally, but was full of brown and fœtid pus. The body of the tumour, by which the fungi were produced, had the appearance of soft spermaceti, and was striated by a vascular substance of a brownish colour. It weighed forty-six ounces. This state of the kidney accounted for the state of the urine mentioned above. The pancreas and mesenteric glands partook of the same state of disease. The lungs were of a milky whiteness, having roundish tumours of various sizes, and of a copper colour spread over the surface, and entering their substance. Externally the appearance of these were, as if a handful of copper coins had been shaken over, and had adhered to the surface of the lungs. These tumours were very hard; and when cut into, appeared more like indurated glands than any thing else.

The pericardium contained no fluid; but this I attributed to the corpse being opened an hour after death. Both the external and internal mammary glands were much enlarged.

The symptoms observed before death, and the appearances on dissection, leave no doubt in my mind of this being a well marked case of that most dreadful of all diseases, Fungus Hæmatoides.

